# Fast Energy Dependent Scatter Correction for List-Mode PET Data

**DOI:** 10.3390/jimaging7100199

**Published:** 2021-09-30

**Authors:** Juan Manuel Álvarez-Gómez, Joaquín Santos-Blasco, Laura Moliner Martínez, María José Rodríguez-Álvarez

**Affiliations:** Instituto de Instrumentación para Imagen Molecular (I3M), Centro Mixto CSIC-Universitat Politècnica de València, 46022 València, Spain; chimosb90@gmail.com (J.S.-B.); lmoliner@i3m.upv.es (L.M.M.); mjrodri@i3m.upv.es (M.J.R.-Á.)

**Keywords:** list-mode, positron emission tomography (PET), energy-based scatter correction, Monte Carlo simulation

## Abstract

Improvements in energy resolution of modern positron emission tomography (PET) detectors have created opportunities to implement energy-based scatter correction algorithms. Here, we use the energy information of auxiliary windows to estimate the scatter component. Our method is directly implemented in an iterative reconstruction algorithm, generating a scatter-corrected image without the need for sinograms. The purpose was to implement a fast energy-based scatter correction method on list-mode PET data, when it was not possible to use an attenuation map as a practical approach for the scatter degradation. The proposed method was evaluated using Monte Carlo simulations of various digital phantoms. It accurately estimated the scatter fraction distribution, and improved the image contrast in the simulated studied cases. We conclude that the proposed scatter correction method could effectively correct the scattered events, including multiple scatters and those originated in sources outside the field of view.

## 1. Introduction

In positron emission tomography (PET), Compton scattering refers to a scenario in which one or both annihilation photons suffer a change in direction and energy when interacting with matter. It is important to compensate Compton scattering in order to enhance the contrast of the reconstructed images and to improve activity quantification.

Among several existing approaches to solve the scatter problem in PET [1,2,3], the most popular one is the single scatter simulation (SSS), in which a scattered image is simulated and incorporated into the reconstruction process [4]. This method is quite accurate, but it requires prior knowledge of the activity distribution, inside the field of view (FOV), combined with an attenuation map of the object. The aforementioned method yields improvable results in cases where multiple scatters are important, such as in the case of whole body PET studies.

Some approaches use time-of-flight (TOF) information [5], attenuation maps [6,7,8,9,10], or a joint activity, i.e., attenuation estimation from positron emission tomography scatter [11]. Nonetheless, our purpose is to obtain a good estimation of scatter without using TOF or attenuation maps.

Another approach to correct scatter degradation consists of Monte Carlo scatter simulation (MCSS) [12]. Monte Carlo applications accurately simulate gamma ray interactions with the detectors. However, the major drawback is simulating the object of the study with high precision, and therefore computed tomography (CT) attenuation maps as input for the simulations are often needed.

Nowadays, there is a general trend to study scatter corrections by applying machine learning techniques [13] that have shown promising results, although more development is needed. All these methods are currently computationally too expensive and time-consuming due to repetitive looping over numerous parameters. Furthermore, they are based on sinogram input data and, in most of the cases, do not take advantage of the energy information.

Energy-dependent scatter methods try to estimate the percentage of events that have suffered Compton interactions based on the recorded energy of those events. These energy approaches have been broadly implemented in single-photon emission computed tomography (SPECT). However, implementations in PET are limited due to the poor energy resolution of the detectors [14] and the predominant Compton interactions taking place in PET scintillators that make it difficult to distinguish whether a photon underwent a scattering interaction in the patient’s body or in the detector.

Among many different energy window-based scatter correction approaches in SPECT, the simplest implementation is the dual energy window (DEW). In this method, γ-rays are acquired within the photopeak and a lower-energy scatter window [15]. The projection profiles obtained in the scatter window are multiplied by an empirically defined weighting factor, and subtracted from the projection files in order to obtain the unscattered projection data [16]. Another approach that falls into this category is the triple energy window, which employs two narrow subwindows on both sides of the photopeak. The scattered photons in the photopeak window are estimated from these subwindows, and then subtracted from the photopeak window, as in the previous DEW method [17].

Nowadays, new scintillator detector systems have been developed for PET scanners that increase energy resolution and detection efficiency. This is due to the use of more appropriate radiation detection materials with higher density and atomic number (Z), as well as replacing the traditional photomultiplier tube (PMT), for more modern silicon photomultiplier (SiPM). A full comparison of these gamma photon detector systems can be found in Matthew Lowdon et al. [18]. Medical imaging based on gamma ray detection takes advantage of those developments; techniques such as PET, SPECT, or Compton cameras have increased the resolution and the contrast of their images. Hybrid scanners combining two of these techniques have also been developed with great success [19,20].

These recent improvements in the detector energy resolution of PET scanners, as well as the capacity of the current detectors to estimate the energy of two photons in coincidence [21,22] with the possibility to store them in list-mode format (not binned into histogram or sinogram) have allowed the implementation of scatter correction based on the analysis of energy spectra [23,24].

In this study, we investigate an energy window approach to correct the scatter component using energy information only. Scatter correction is one of the major challenges for accurate quantification in 3D PET, in particular, if the scatter is due to activity originating from outside the field of view [25]. We employ list-mode PET data to compensate the several limitations present in other approximations, such as not being able to estimate the scatter component originated by the activity outside the FOV.

Our energy scatter correction algorithm is similar to the ones previously proposed by Chen et al. [26] and by Popescu et al. [27]. In our approach, the introduction of energy-dependent weights is needed before reconstruction, thus, data preprocessing is necessary. However, once this previous step is completed, our method requires minimal computation time for image reconstruction, and therefore is appealing for practical use.

The principal aim of this study is to implement a fast, simple and practical energy-based scatter correction method which uses predefined weighted factors and to study its accuracy through a Monte Carlo simulation test.

## 2. Materials and Methods

List-mode format files normally store the information related to the impacts of two photons corresponding to an event. In particular, the impact position, the detector identifier, the time stamp, and the amount of energy deposited in the detectors by these photons are the required inputs for this method. Our method consists of a preprocessing of the energy information stored in list-mode file, in order to reduce the scatter effect, similar to the work of B. Guerin and G. El Fakhri [28] but avoiding the need to order our file in angular projections to generate a sinogram file. 

**Figure 1:**
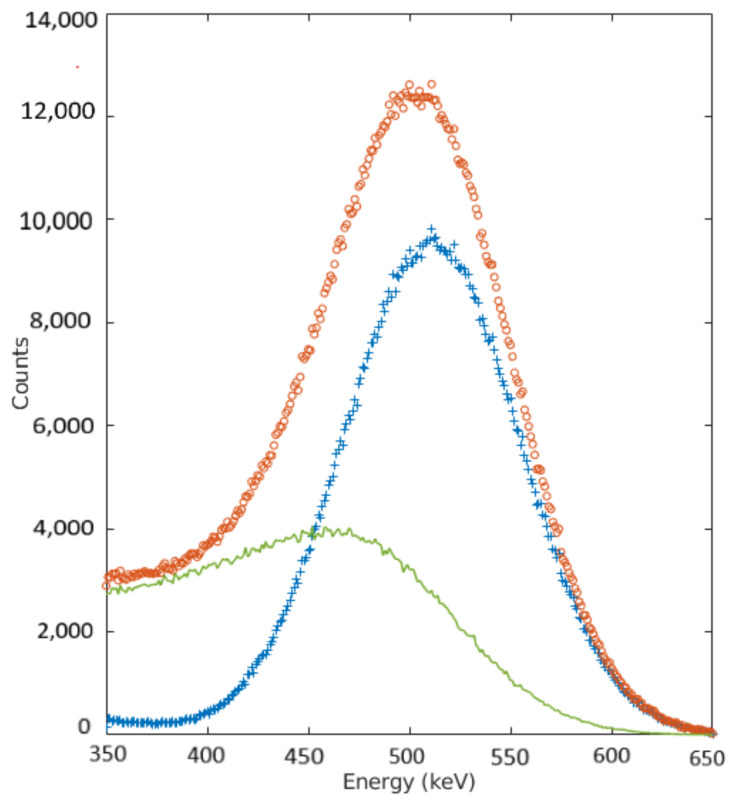
Energy distribution of the registered events from a 16 cm radius water-filled cylinder with a line source in the center. Total spectrum (empty dots), trues (crosses), and scatter (solid line). The scatter and true contributions to the total spectrum can be appreciated.

Our approach exploits the fact that a high proportion of the scattered photons recorded in the energy spectrum are set below the photopeak region as compared with those recorded near the photopeak, as it shown in Figure 1.

Thus, the data recorded in the energy windows below or above the photopeak window, or both, can be used to derive an estimation of the scatter contribution through scaling factors. They account for the different sensitivity of the scattered events in the total and auxiliary windows, and they are derived from simulations of several attenuation media before reconstruction [29]. In the following subsections, we describe how to apply these corrections to a PET image reconstruction process.

### 2.1. Scanner Design and Simulation Setup 

System simulations were performed using the Monte Carlo package GATE (Geant4 Application for Tomographic Emission) [30]. In our simulations, the MINDView PET geometry as well as the system parameters were modeled through a handmade GATE macro script. MINDView is a dedicated brain PET scanner composed of one ring of 20 detector blocks [31]. Each detector block includes a thick monolithic LYSO (Lutetium-yttrium oxyorthosilicate) crystal with dimensions of 50 × 50 × 20 ${\mathrm{mm}}^{3}$. Each crystal is coupled to a custom array of 12 × 12 SensL SiPM [32]. The axial FOV is 60 mm and the transaxial FOV is 330 mm. This PET system has an overall energy resolution of ~10% with precise depth of interaction (DOI) estimation capability which is incorporated into the list-mode file [32]. To calculate the energy resolution of the system an array of N 22a was used, as described in Gonzalez et al. [33]. The energy resolution for each detector block at 511 keV was determined by fitting a Gaussian curve to energy histogram. The percentage (10%) was calculated as the ratio of full width at half maximum (FWHM) to the centroid value.

Using GATE, it is possible to simulate the random process of emission and detection of gamma photons in a PET system. The following physical processes were included in the simulations: Compton scattering, Rayleigh scattering, electron ionization, and photoelectric interaction. We chose F 18 sources, an acquisition time of 60 s, a 10% energy resolution, and 350–650 keV as the energy cut set in every performed simulation. The results from Figure 1 correspond to the energy spectra of the positron line source in the center of the FOV, placed inside a water cylinder phantom. We included unscattered events as well as photons scattered at least once within the phantom in order to characterize the energy spectrum of our system.

### 2.2. Choice of Energy Windows

Two configurations of auxiliary energy window were selected after several simulation tests. 

#### 2.2.1. Double Energy Window Method

A low energy window (LEW) (350–430 keV) and a photopeak energy window (430–650 keV) were chosen. The scatter events on the photopeak region $C_{ph_{scatter}\;\left(i\right)}$ were calculated as:

(1) $$C_{ph_{scatter}\;\left(i\right)}=k_{LEW}\times C_{LEW\;\left(i\right)}$$

where $C_{LEW\;\left(i\right)}$ are the counts recorded in the LEW for the *i*th line of response (LOR), and $k_{LEW}$ is defined as the quotient between the scattered events in the photopeak window and the number of events in the low energy window.

#### 2.2.2. Triple Energy Window Method

A low energy window (LEW) (350–430 keV), a photopeak energy window (430–550 keV) and an upper energy window (UEW) (550–650 keV) were selected. We assumed that most of the counts in the upper energy window had not suffered scatter, as ascertained from Figure 1. The scatter component in the photopeak region was calculated using these windows as:

(2) $$C_{ph_{scatter}\;\left(i\right)}=k_{LEW}\times C_{LEW\;\left(i\right)}+k_{HEW}\times C_{HEW\left(i\right)}$$

where $C_{LEW\;\left(i\right)}$ and $C_{HEW\left(i\right)}$ are the number of counts recorded in the low and the upper energy windows; $k_{LEW}$ and $k_{HEW}$ are defined as the quotient between the scattered events in the photopeak window and the number of events recorded in the low energy window and the high energy window, respectively.

### 2.3. Scatter Algorithm

**Figure 2:**
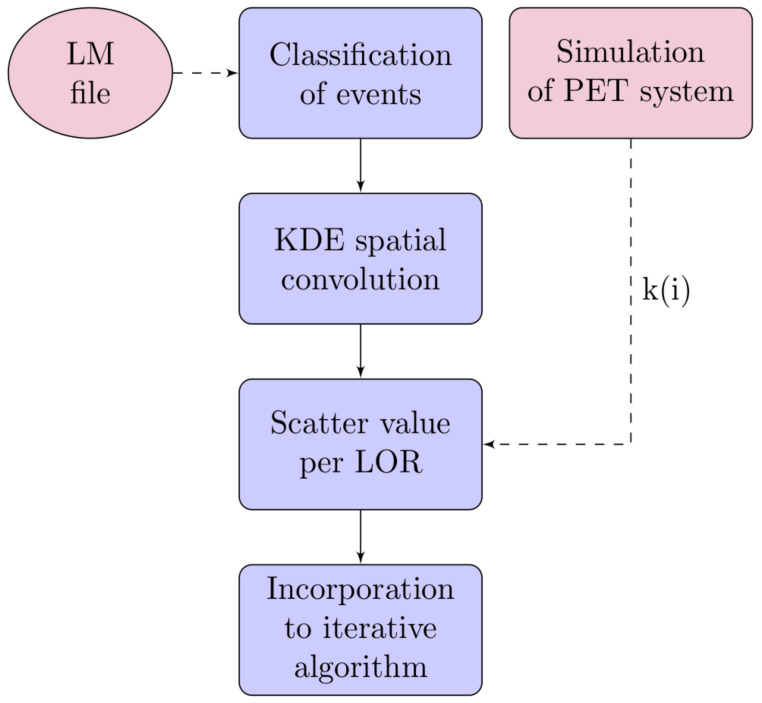
Flowchart with the steps involved in the proposed scatter algorithm.

Four steps are involved in the proposed scatter correction algorithm (Figure 2), as follows:

#### 2.3.1. Simulation of the PET System and Calculation of the Scatter Coefficients

The estimation of the scatter coefficients of Equations (1) and (2) were computed using the data of the GATE simulation of the scanner, depending on the window configuration. Considering a window w, the corresponding scatter coefficient is defined by the fraction:

(3) $$k_{w}=\frac{C_{ph\;scatter}^{GATE}}{C_{w}^{GATE}},$$

where $C_{ph\;scatter}^{GATE}$ is the total number of scattered counts and $C_{w}^{GATE}$ are the scattered counts simulated in the auxiliary window. Thereby, the total number of true counts in the photopeak region can be derived as:

(4) $$C_{ph\;true}^{GATE}=C_{ph\;total}^{GATE}-\underset{w}{\sum }k_{w}\times C_{w}^{GATE}$$

where the sum applies for all the windows in the selected configuration.

Simulated data are necessary for the computation of the model parameters and the contour conditions, i.e., the correspondence between the fraction of scatter events and those registered in the auxiliary windows. Due to the energetic resolution of our system, it may happen that some photons are collected in the wrong energy window. When the simulations are performed, it is important to model the energy resolution of our system, and therefore, in this way, the introduced coefficients can partially mitigate this effect.

To incorporate scatter correction into the iterative reconstruction, the expected scatter value per LOR is needed [34]. However, it might happen that an estimation method provides an accurate value of the total number of scatter counts, but, at the same time, a very poor estimation of the number of scatter events in each LOR. Such estimates would constitute an extra source of image degradation. Furthermore, a typical PET acquisition contains fewer coincidences than the number of all possible LORs, and therefore additional data preprocessing is necessary to compensate those regions of the FOV with low statistics.

#### 2.3.2. Storing and Processing Events from the List-Mode Input File

The next step of the method is to classify the list-mode events according to their energy. A list-mode event (or count) is parameterized by the energies of the pair of measured photons and the positions on their respective detector blocks. Two photons with energies within the photopeak window determine a photopeak event, and to record an event in an auxiliary window, one of the detected photons must be in the energy range of that window. Adjacent LORs are grouped in bins according to a user-defined final image resolution, i.e., a given LOR bin (or bin) contains all the counts within two specific pixels of a faced pair of detector blocks. Considering that the total number of registered events depend on the sample activity and the selected LOR bin, the distribution of counts among the energy windows is often distorted and statistically non-significant. This problem is further accentuated in those FOV regions with fewer events. To overcome this misrepresentation, the count pattern distribution for a given bin also considers the events registered by its adjacent bins. For this purpose, a spatial convolution of the list-mode file using a multivariate kernel density estimate (KDE) is proposed [35,36]. Thus, the counts obtained for a given (center) bin are computed as the sum of those measured on its neighborhood, weighted by a proper function (kernel) that accounts for the proximity between the adjacent and the center bins.

The convolution is performed independently for the different energy windows and a standard Gaussian kernel is employed. Given two events with LOR spatial coordinate vectors (i.e., the four vectors constituted by the pair of spatial coordinates of the two involved detectors) *x* and $x_{0}$, the weight for the counting of the first one on the latter is:

(5) $$\omega \left(\Delta_{x}\right)\;=N\;e^{-\frac{1}{2}\Delta x^{t}H\Delta x}$$

where $\Delta x=x-x_{0}$; N is a normalization factor; and H is a positive definite symmetric 4 × 4 matrix, so-called the bandwidth matrix, that parameterizes the geometry of the LOR neighborhood. The optimal value for H is computed as the one which minimizes the mean integrated squared error for a given distribution. A good approximation, however, is provided by Silverman’s rule of thumb:

(6) Hii=23n14σi2;  Hij=0

where *n* is the number of counts and $\sigma_{i}^{\;}$ is the standard deviation of the *i*th LOR spatial coordinate [37]. A cut-off on the neighborhood size has also been applied (95% kernel volume contour).

After this preprocessing, an accurate estimation of the counts of each energy window is obtained for each bin.

#### 2.3.3. Scatter Fraction Estimation per LOR

The coefficients previously calculated from simulations, along with the number of events in each energy window, were used to determine the scatter counts in the photopeak region for each LOR bin. The addition of this value to the LEW event contributions determines the total number of scatter counts present in each bin. Considering that the fraction of scattered events remains constant for the LORs creating a bin, the scatter fraction per LOR $\left(SF_{i}\right)$ can be calculated as:

(7) $$SF_{i}=\frac{\sum_{i}^{\;}scatter_{i}}{\sum_{i}^{\;}true_{i}+scatter_{i}}=\frac{C_{LEW\;\left(i\right)}+C_{ph_{scatter}\;\left(i\right)}}{C_{Total\left(i\right)}}$$

This scatter value provides an estimation of the probability that an event detected along the *i*th LOR is a scatter event.

#### 2.3.4. Suppression of the Scatter Counts and Validation

The scatter correction estimation was incorporated during the iterative reconstruction using maximum likelihood expectation maximization (MLEM) [38]:

(8) $$n_{j}^{k+1}=\frac{n_{j}^{k}}{\sum_{i=1}^{I}a_{ij}}\overset{M}{\underset{i=1}{\sum }}a_{ij}\frac{1}{\sum_{j=1}^{J}a_{ij}n_{j}^{k}+S_{i}}$$

where $n_{j}^{k}$ represents the *j*th voxel value of the image (at the *k*th iteration), $a_{ij}$ is the probability of an emission from the *j*th voxel being detected along the *i*th LOR, the $a_{ij}$ coefficients also include attenuation and detector normalization, M is the number of measured events, I is the number of all possible system LORs, and $S_{i}$ is the scatter estimation for the *i*th LOR. This $S_{i}$ factor is added as a constant term at each iteration of the forward model [2].

The conventional approach is to subtract the scatter estimation data from the noisy measured projections before the reconstruction procedure. Newly developed methods use the scatter component as a constant additive term with the forward model in the reconstruction loop [39]. This approach shows better noise properties as compared with the subtraction or the fully precorrection methods [40].

## 3. Validation and Results

To evaluate the proposed scatter correction method, a series of Monte Carlo simulations were performed using the numerical simulation software GATE [41], which has been widely used in research studies showing good agreement with experimental data [30]. The MINDView PET system was modeled using this tool. These Monte Carlo simulations use the appropriate energy-dependent cross-sections for the interaction of photons in water and in the LYSO detector.

Scatter events in the phantom can be tracked and stored in a list-mode file output (ASCII or ROOT output [42]). By performing these simulations, we optimized the energy windows, determined the scatter in the different energy windows, and made an estimation of the scatter coefficients of our system, as described in Section 2.3. The scatter ratios obtained in each energy window are presented in Table 1.

When using MonteCarlo simulations, it is possible to identify the true distribution of scatter photons along the FOV; thus, the correct scatter fraction can be extracted from simulations. Although such an ideal estimation method is not possible in real acquisitions, it allows us to isolate the degradation effects due to scatter and to determine the maximum gain achievable at the reconstructed image level. From the simulations, the $k_{w}$ values were calculated in each bin of the FOV in our PET scanner.

**Figure 3:**
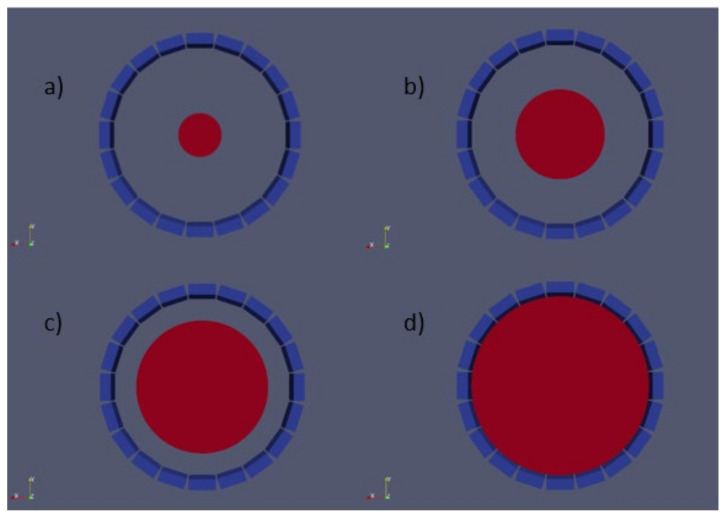
Simulation of a water cylinder with different radii and the same activity source. It can be appreciated the detector system and the phantoms used. (**a**–**d**) 40, 80, 120, and 160 mm radius.

Seven water-filled cylinder phantoms were simulated with 60 mm of height and a radius ranging from 40 to 160 mm, as shown in Figure 3, to cover all the FOVs of our PET system. A line source of 5 × 60 mm^2^ with 10 MBq of activity was placed in the center for each phantom. 

Then, we extracted the necessary information from the GATE files and generated our own list-mode files from the simulations. We used these files as the input for our reconstruction algorithm and made an estimation of the scatter fraction, first, using only the information of a low energy window and, second, using both low and upper energy windows. Table 2 shows the scatter fraction obtained for each case.

We also compared the performance of our proposed algorithm with the standard single scatter simulation method (SSS). We used a model-based single scatter simulation algorithm, which was implemented in an open-source software library called Software for Tomographic Image Reconstruction (STIR) [43]. A simulation of our MINDView PET system was incorporated into STIR, along with the attenuation region known through our Monte Carlo simulation. Scatter points are selected only if the attenuation value of a particular voxel is above a given threshold, in our case, inside the water cylinder.

We used the same simulated setup as before, i.e., seven water-filled cylinder phantoms with 60 mm of height and radius ranging from 40 to 160 mm. After obtaining the corrected sinogram, we isolated and compared the estimated number of scattered events and calculated a scatter fraction. The results obtained can be seen in Table 3 together with the results of the low-upper window method results.

**Figure 4:**
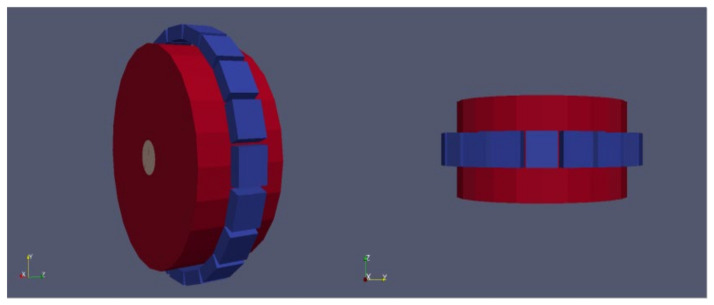
Simulation of a cylindrical source inside a water cylinder 25 mm radius × 160 mm height. The height of the phantom and the source are larger than the height of the FOV of the simulated scanner (50 mm).

To test the proposed algorithm with sources out of the FOV, a series of simulations with a line uniform source of 25 mm radius × 160 mm height inside a water cylinder of 120 mm radius × 160 mm height were performed (Figure 4).

In addition, to validate the algorithm for different numbers of registered events, we repeated the same simulation setup with different activity sources. Table 4 shows the scatter fractions obtained in each case. A better performance is achieved when the upper energy window information is incorporated. 

Around 30% of the simulated scatter events come from decays outside the FOV, our proposed method accurately corrects these events due to energy discrimination, as can be seen in Table 4.

**Figure 5:**
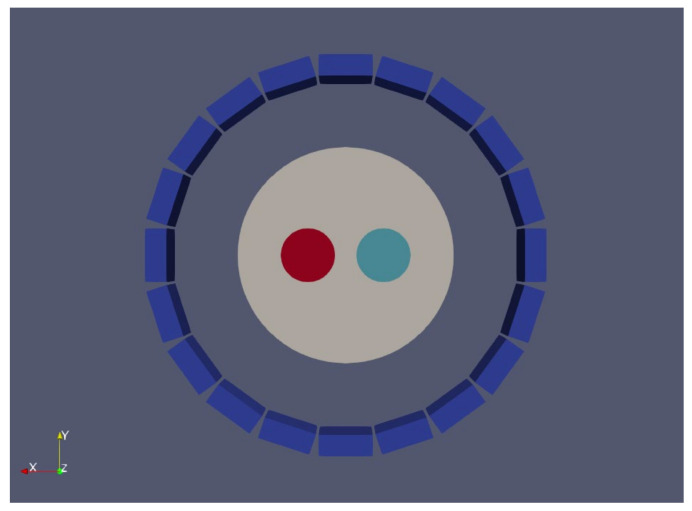
Simulation of Utah phantom. A rod with 10:1 activity (left, red) with respect to the external cylinder and a rod without activity (right, cyan) were simulated.

Finally, to study the spatial dependence of our scatter method, a Utah phantom [44] was also simulated. The phantom was composed of an external cylinder (100 mm radius × 50 mm height) and two rods (25 mm radius × 50 mm height) separated 35 mm from the center of the external cylinder. One of the rods had no activity and the other had a 10:1 activity ratio as compared with the external cylinder (Figure 5).

We implemented the scatter correction method in the iterative algorithm (MLEM) as well as its accelerated variant with ordered subsets (OSEM) [45]. The purpose of using both algorithms was to check that there was no bias regarding the reconstruction method.

**Figure 6:**
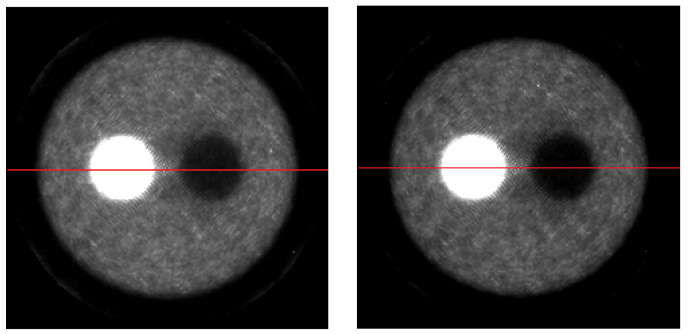
Reconstructed images of the Utah phantom using LM-OS with 4 iterations and 3 subiterations: (**left image**) Without scatter correction; (**right image**) scatter corrected using the information of the upper and low energy windows (TEW method).

**Figure 7:**
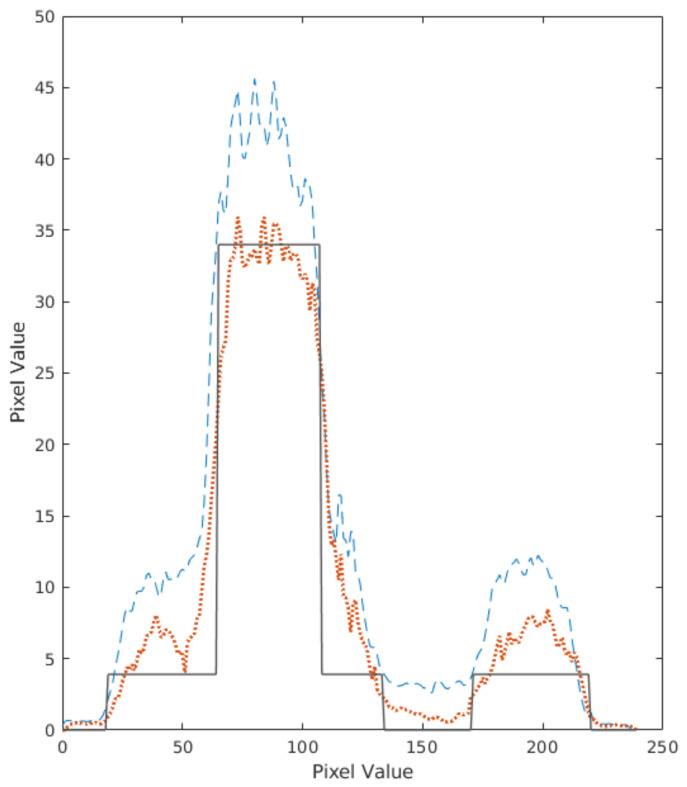
Spatial distribution of counts in a central slice of the reconstructed Utah phantom images, without scatter correction (dashed line), scatter corrected (dots) and generated ground truth using MC simulation (solid line). Vertical pixel value refers to the grey intensity scale.

In every iteration, a sensitivity correction was applied. Virtual detector pixels, in the range 1.4–2 mm, were tested, combined with voxel sizes of 1–2 mm. The reconstructed images of the Utah phantom are shown in Figure 6. To correct scatter, the triple energy window method was used. The corrected and uncorrected profiles of the center image of the phantom are shown in Figure 7.

## 4. Discussion

The proposed method uses energy information to locally estimate the right proportion of scatter events in PET data for all FOV regions. The employed energy-based window approach succeeds in suppressing up to 90% of the scatter component using optimized factors according to the selection of energy windows here presented. Two configurations are analyzed in this study: the double energy window, with a low energy window below the photopeak energy region, and the triple energy window, which also incorporates a high energy window above the photopeak. Our method exhibits good agreement with the Monte Carlo simulation data and improves the contrast of reconstructed images of a Utah phantom. Furthermore, a better agreement with simulation data is achieved once the information of the upper energy window is incorporated.

One of the advantages of this energy-based method is the use of list-mode PET data, instead of sinogram information, and therefore it can operate at the LOR scale, i.e., the scatter fraction is evaluated for each LOR. In addition, it can be adjusted to a user-defined energy and spatial resolution by means of conveniently binning the data. To compensate those sources with low activities, small-sized attenuation media or FOV regions with low count-rate statistics, a convolution of the list-mode file is proposed. That is, the calculation of the scatter fraction of a given LOR not only considers the events registered in its corresponding detectors, but also those placed nearby, provided an appropriate weight function is applied. Consequently, the method can predict true and scatter events in all FOV regions, and its count-rate dependency is palliated. Despite correcting the cases studied with few events, we have still found discrepancies (~2%) with the simulated data in cases with high activity, as can be seen in Table 4. This is ascribed to an overestimation of the counts in the lower energy window in the bins between detectors farthest from the center of the FOV.

Our proposed method also exhibits better agreement with simulated data than the tested version of the SSS algorithm. The SSS method underestimates the number of scatter events. This may be due to the fact that multiple scatters are not included in this model, and therefore more scatter points need to be incorporated in the simulation. The SSS model is also more time-consuming than our method, once the MC simulated is done, we need to identify the attenuation medium and assign scatter points. Furthermore, we must organize the collected data into projections, this step must be undone before the reconstruction process. In contrast, our energy window method can work directly with raw data, once the phantom is simulated, the only time-consuming section is the convolution part of the file. Although further investigation is needed, we have shown that our energy window approach may be a good alternative to sinogram-based scatter algorithms.

We presented a basic approach to a complex, multi-dependent problem, with the advantages of being fast, easy to implement, and with minimal computation expense in the reconstruction procedure. Our method also takes into consideration the scatter arising from activity beyond the axial FOV, as can be seen in Table 4, and works directly from list-mode format files.

We have shown that the energy window technique described in this study may provide a practical, accurate, and a general-purpose choice for correcting scatter in 3D PET. Our method corrects the scatter degradation without requiring a prior attenuation map of the studied object. Finally, we conclude that the scatter estimation of this method is in agreement with Monte Carlo simulated data, and yields good results for symmetrical objects with an approximately homogeneous activity distribution, such as is the case for dedicated brain PET systems.

## Figures and Tables

**Table 1:** Ratio of scatter-true events for each energy window (water-filled cylinder 16 cm).

Energy Window (keV)	350–430	430–550	550–650
True events	14.34%	68.86%	89.73%
Scatter events	85.66%	31.14%	10.27%

**Table 2:** Scatter fraction obtained by Monte Carlo simulations and by the proposed method. A water cylinder with different radii and the same activity source was simulated.

Radius Phantom (mm)	SF Monte Carlo	SF Low Window	SF Low-Upper Window
40	0.127	0.179	0.142
60	0.182	0.210	0.193
80	0.236	0.254	0.249
100	0.318	0.353	0.342
120	0.377	0.402	0.379
140	0.454	0.475	0.465
160	0.532	0.571	0.526

**Table 3:** Scatter fraction obtained by Monte Carlo simulations, by SSS method, and by the proposed method. A water cylinder with different radii and the same activity source was simulated.

Radius Phantom (mm)	SF Monte-Carlo	SF (SSS)	SF Low-Upper Window
40	0.127	0.103	0.142
60	0.182	0.169	0.193
80	0.236	0.208	0.249
100	0.318	0.292	0.342
120	0.377	0.350	0.379
140	0.454	0.417	0.465
160	0.532	0.501	0.526

**Table 4:** Scatter fraction obtained by Monte Carlo simulations and by the proposed method. A water cylinder and a line source larger than the FoV were simulated.

Activity (MBq)	SF Monte-Carlo	SF Low Window	SF Low-Upper Window
10	0.729	0.659	0.696
30	0.728	0.678	0.709
50	0.728	0.681	0.725
80	0.727	0.705	0.730
100	0.728	0.735	0.734
130	0.721	0.772	0.739
150	0.724	0.792	0.741

## Data Availability

Not applicable.
